# Drought stress enhances nutritional and bioactive compounds, phenolic acids and antioxidant capacity of *Amaranthus* leafy vegetable

**DOI:** 10.1186/s12870-018-1484-1

**Published:** 2018-10-26

**Authors:** Umakanta Sarker, Shinya Oba

**Affiliations:** 1 The United Graduate School of Agricultural Science, Laboratory of Field Science, Faculty of Applied Biological Sciences, Gifu University, Yanagido 1-1, Gifu, Japan; 2 Department of Genetics and Plant Breeding, Faculty of Agriculture, Bangabandhu Sheikh Mujibur Rahman Agricultural University, Gazipur, 1706 Bangladesh

**Keywords:** *Amaranthus tricolor*, Nutritional and bioactive compounds, Phenolics, Flavonoids, Antioxidant activity, HPLC-UV, LC-MS-ESI, DPPH, ABTS+, Drought

## Abstract

**Background:**

Bioactive compounds, vitamins, phenolic acids, flavonoids of *A. tricolor* are the sources of natural antioxidant that had a great importance for the food industry as these detoxify ROS in the human body. These natural antioxidants protect human from many diseases such as cancer, arthritis, emphysema, retinopathy, neuro-degenerative cardiovascular diseases, atherosclerosis and cataracts. Moreover, previous literature has shown that drought stress elevated bioactive compounds, vitamins, phenolics, flavonoids and antioxidant activity in many leafy vegetables. Hence, we study the nutritional and bioactive compounds, phenolic acids, flavonoids and antioxidant capacity of amaranth under drought stress for evaluation of the significant contribution of these compounds in the human diet.

**Results:**

The genotype VA3 was assessed at four drought stress levels that significantly affected nutritional and bioactive compounds, phenolic acids, flavonoids and antioxidant capacity. Protein, ash, energy, dietary fiber, Ca, K, Cu, S, Mg, Mn, Mo, Na, B content, total carotenoids, TFC, vitamin C, TPC, TAC (DPPH), betacarotene, TAC (ABTS^+^), sixteen phenolic acids and flavonoids were remarkably increased with the severity of drought stress. At moderate and severe drought stress conditions, the increments of all these components were more preponderant. *Trans*-cinnamic acid was newly identified phenolic acid in *A. tricolor*. Salicylic acid, vanilic acid, gallic acid, chlorogenic acid, *Trans*-cinnamic acid, rutin, isoquercetin, *m*-coumaric acid and *p*-hydroxybenzoic acid were the most abundant phenolic compounds in this genotype.

**Conclusions:**

In *A. tricolor,* drought stress enhanced the quantitative and qualitative improvement of nutritional and bioactive compounds, phenolic acids, flavonoids and antioxidants. Hence, farmers of semi-arid and dry areas of the world could be able to grow amaranth as a substitute crop.

## Background

Both researchers and consumers have much interests to natural antioxidants of vegetables. These natural compounds protect many diseases, such as cancer, arthritis, emphysema, retinopathy, neuro-degenerative and cardiovascular diseases, atherosclerosis, and cataracts [1–4]. *Amaranthus tricolor* is an inexpensive and excellent source of lots of natural antioxidants like nutritional and bioactive compounds, phenolics, flavonoids and detoxifies reactive oxygen species (ROS) in human body [2, 5].

The intensity of damage caused by reactive oxygen species (ROS) mainly depends on its balance between production and elimination by the antioxidant scavenging system [6]. Moreover, drought stress favors rapid damage and leakage of plant cell membrane [6]. Environmental stresses cause oxidative damage in the plant. Stressed-plants have also protection systems to overcome the oxidative damage by synthesis of secondary metabolites like phenolics, flavonoids [7, 8]. These compounds can detoxify ROS in plants, and also have the capacity to cure many human diseases caused by oxidative damage and aging [9].

Amaranths can tolerate drought efficiently [10, 11]*. A. tricolor* is a well-acclimated leafy vegetable against biotic and abiotic stresses [12] and had multipurpose usages. Many processes, such as environmental, biological, ecological, physiological, biochemical and evolutionary process are involved in the quantitative and qualitative improvement of natural antioxidants of this species of which, drought stress can rapidly boost up the contents [13].

There is limited information on leafy vegetables regarding the effect of secondary metabolites to drought stress, such as nutritional and bioactive compounds, phenolics, flavonoids and antioxidants. Drought stress enhanced secondary metabolites, such as betacarotene composition in Choysum varieties [14] and in perennial herbaceous [15], vitamin C in tomato [16], total polyphenol and total flavonoid content in buckwheat [17], total antioxidant activity, total polyphenol and total flavonoid content in *Achillea* species [18]. On the other hand, drought stress declined buckwheat’s protein composition [17], betacarotene composition of Kailaan variety [14] and vitamin C, Zn, Ca and Fe content of both varieties [14]. There is no literature regarding drought stress effects on nutritional and bioactive compounds, phenolics, flavonoids and antioxidant activity in *A. tricolor*. Our earlier studies [19–26], we selected some antioxidants enriched and high yielding genotypes. Consequently, the study was aimed to evaluate the drought stress effects on selected genotype for nutritional and bioactive compounds, phenolics, flavonoids and antioxidant activity.

## Methods

### Experimental site, plant materials and experimental conditions

Earlier, we collected 102 genotypes in different eco-geographical zones of the country. From this collection, an antioxidant enriched high yield potential genotype (Accession VA3) was selected based on our previous studies [19–26]. This genotype was grown in pots under rain shelter open field of Bangabandhu Sheikh Mujibur Rahman Agricultural University, Bangladesh (AEZ-28, 24^0^23’ north latitude, 90^0^08’ east longitude, 8.4 m.s.l.). 22 cm height and 24 cm diameter (upper side) plastic pots were used to raise the seedlings. For pots preparation, soil and cow dung were mixed @ 2:1 ratio. For proper germination and dynamic seedling growth, pots were properly irrigated every day up to 10 days after sowing of seeds (DAS). At first, field capacity of soil used in pots was measured by the gravimetric method. Then the amount of water at 100% field capacity was measured by subtracting the weight of completely dry soil from the weight of soil at 100% field capacity. Pot weight (including pot soil) for each treatment was calculated by weighing of completely dry soil and amount of water required for attaining respective field capacity. Pots were irrigated at 100% field capacity up to 5 days after planting (DAP) for dynamic growth and proper establishment of seedlings. After establishment period, *A. tricolor* plants were subjected to the different irrigation treatments as FC (100% field capacity, control), mild stress (90% FC), moderate stress (60% FC), and severe stress (30% FC). Throughout cultivation period, moisture levels in the soil were controlled by daily weighting following the standard procedure. Pots were weighed twice a day at 12 h intervals. To achieve the target field capacity of each water condition, the amount of water equaling that lost through transpiration and soil evaporation, percolation and leaching were added. Imposition of water stress was continued up to 30 DAP. At 30 DAP the leaves of *A. tricolor* were harvested from each experimental unit. All the parameters were measured in three replicates.

### Chemicals

Solvent: methanol and acetone. Reagents: Standard compounds of pure phenolic acids, HPLC grade acetonitrile and acetic acid, vitamin C, gallic acid, rutin, methanol, DPPH (2,2-diphenyl-1-picryl-hydrazyl), ABTS^+^(2,2-azinobis-3-ethyl-enzothiazoline-6-sulphonicacid), trolox (6-hydroxy-2,5,7,8-tetra-methyl-chroman-2-carboxylicacid), aluminum chloride hexa-hydrate, sodium carbonate, potassium acetate, Folin-Ciocalteu reagent, H_2_SO_4_, NaOH, HNO_3_, HClO_4_, lanthamum, Caesium chloride, dithiothreitol (DTT) and potassium persulfate. The pure and analytical grade solvents and reagents from Kanto Chemical Co. Inc. (Tokyo, Japan) and Merck (Germany) were used in this experiment.

### Proximate composition

ASAE standards were followed to determine the moisture content [27]. *A. tricolor* leaves were dried in an oven at 103 °C for 72 h. The dried leaves were then transferred to a desiccator for cooling at room temperature. The leaves were then weighted in a digital balance (Denver Instruments, Denver, Colorado, USA) to record the sample weight.

AOAC methods were followed to measure ash, crude fat, and crude protein contents [28]. Leaf samples were weighed before and after heat treatment (550 °C for 12 h) to measure the ash content. AOAC method 960.39 was followed to measure the crude fat content.

Total nitrogen content was measured according to the micro-Kjeldahl method. Finally, total nitrogen was converted to crude protein multiplied by the factor 6.25 (AOAC method 976.05). Fiber was determined by ISO method 5498 [29]. At first, 0.255 M sulfuric acid was added to the leaf powder sample and boiled for 30 min. The insoluble residue was filtered, washed, and boiled in 0.313 M sodium hydroxide. Finally, it was dried at 130 ± 2 °C for 2 h. At 350 ± 25 °C temperature and the weight loss was determined. Fiber content was measured relative to the fresh weight (FW). By subtracting the sum of percent moisture, ash, crude fat, and crude protein from 100, carbohydrate content (g 100 g^− 1^ FW) was measured. A bomb calorimeter was used to determine gross energy following ISO method 9831.

### Estimation of mineral content

At first, *A. tricolor* leaves were dried in a well-ventilated oven at 60 °C for 24 h. Dried leaves were ground finely in a mill. Nitric-perchloric acid digestion methods were used to determine the minerals contents, such as Ca, Mg K, P, S, Fe, Mn, Cu, Zn, Na, Mo and B from powdered leaves [30]. For nitric-perchloric acid digestion, 0.5 g dried leaves were added to 400 ml of nitric acid (65%) with 40 ml of perchloric acid (70%) and 10 ml of sulphuric acid (96%) in the presence of carborundum beads. After digestion, P was measured by appropriately diluting the solution in triplicate following vitamin C method. Addition of vitamin C and Sb, yellow-colored complex converted to a blue-colored phosphomolybdenum complex. Absorbance was taken according to the method described by Sarker & Oba [31] at wavelength of 880 nm (P), 766.5 nm (K), 422.7 nm (Ca), 285.2 nm (Mg), 258.056 nm (S), 248.3 nm (Fe), 279.5 nm (Mn), 324.8 nm (Cu), 213.9 nm (Zn), 589.0 nm (Na), 313.3 nm (Mo) and 430 nm (B) by atomic absorption spectrophotometry (AAS) (Hitachi, Tokyo, Japan).

### Estimation of betacyanin and betaxanthin content

Fresh amaranth leaves were used to extract betacyanin and betaxanthin using 80% methanol containing 50 mM vitamin C according to Sarker & Oba [31]. Hitachi U1800 spectrophotometer (Hitachi, Tokyo, Japan) was used to measure the absorbance of betacyanin and betaxanthin at 540 and 475 nm, respectively. The mean molar extinction coefficients for betacyanin and betaxanthin were 62 × 10^6^ cm^2^ mol^− 1^ and 48 × 10^6^ cm^2^ mol^− 1^ respectively. Data were recorded as ng betanin g^− 1^of leaves fresh weight for betacyanin and ng indicaxanthin g^− 1^ of *A. tricolor* leaves fresh weight for betaxanthin.

### Estimation of chlorophyll and total carotenoids

The fresh *A. tricolor* leaves were used to estimate chlorophylls and total carotenoids using 80% acetone following Sarker & Oba’s [32] method. Hitachi U1800 spectrophotometer (Hitachi, Tokyo, Japan) was used to read the absorbance at 663, 646 and 470 nm for chlorophyll *a*, chlorophyll *b* and total carotenoids, respectively. Chlorophylls were measured as μg per g fresh weight and carotene was measured as mg total carotenoids per 100 g fresh weight.

### Estimation of betacarotene

Betacarotene were measured following the method Sarker & Oba [31]. 500 mg of fresh leaves were ground in 10 ml of 80% acetone. The extracts were centrifuged at 10,000 rpm for 3–4 min. Removing the supernatant, 20 ml was transported to a volumetric flask. Hitachi U1800 spectrophotometer (Hitachi, Tokyo, Japan) was used to read the absorbance at 510 nm and 480 nm, respectively. Results were recorded as mg betacarotene per g fresh weight.

The betacarotene content was calculated using the following formula:

$$ \mathrm{Amount}\ \mathrm{of}\kern0.17em \mathrm{betacarotene}=7.6\left(\mathrm{Abs}.\mathrm{at}\ 480\right)\hbox{-} 1.49\left(\mathrm{Abs}.\mathrm{at}\ 510\right)\kern0.35em \times \mathrm{Final}\ \mathrm{volume}/\left(1000\times \mathrm{fresh}\ \mathrm{weight}\ \mathrm{of}\ \mathrm{leaf}\kern0.17em \mathrm{taken}\right) $$

### Determination of vitamin C

Vitamin C (AsA) and dehydroascorbate (DHA) acid were measured spectrophotometrically. Dithiothreitol (DTT) was used to pre-incubate the sample for reduction of DHA into AsA. Fe_3_^+^ were reduced to Fe_2_^+^ by AsA and the spectrophotometric (Hitachi, U-1800, Tokyo, Japan) determination of AsA was performed by measuring Fe_2_^+^ complexes with 2, 2-dipyridyl [31]. Hitachi U1800 spectrophotometer (Hitachi, Tokyo, Japan) was used to read the absorbance of the sample solution at 525 nm. Results were recorded as mg vitamin C per 100 g fresh weight.

### Extraction of samples for total polyphenol, flavonoid content and antioxidant activity analysis

At the edible stage (35 days after sowing) leaves were picked and dried in air (In shade) for total polyphenol content, total antioxidant activity and total flavonoid content analysis. 1 g of air-dried leaves from each treatment was ground and extracted in 40 ml of 90% aqueous methanol in a tightly capped bottle (100 ml). The bottle was then placed in a shaking water bath (Thomastant T-N22S, Thomas Kagaku Co. Ltd., Japan) for 1 h. For analysis of total polyphenol content, total antioxidant activity and total flavonoid content the extract was then filtered and stored.

### Estimation of total polyphenol content (TPC)

The Folin-Ciocalteu reagent method described by Sarker & Oba [31] was used to measure the total phenolic content of amaranth leaves. Gallic acid was used as a standard phenolic compound. 1 ml of Folin-Ciocalteu reagent (previously diluted 1:4, reagent: distilled water) and 50 μl of the leaf extract were placed in a test tube, finally mixed thoroughly and allowed to stand for 3 min. Then, 1 ml of Na_2_CO_3_ (10%) was added, and the mixture allowed to stand for 1 h in the dark. Hitachi U1800 spectrophotometer (Hitachi, Tokyo, Japan) was used to read the absorbance at 760 nm. A standard gallic acid graph was used as a standard reference to estimate the total polyphenol content in the leaf extracts. The results are recorded as μg gallic acid equivalent (GAE) g^− 1^ dw.

### Estimation of total flavonoid content (TFC)

Aluminum chloride colorimetric method described by Sarker & Oba [31] was used to estimate the total flavonoid content in leaves extract. 1.5 ml of methanol, 0.1 ml of 1 M potassium acetate, 0.1 ml of 10% aluminum chloride, 500 μl of leaf extract and 2.8 ml of distilled water were transferred to a test tube and allowed to stand for 30 min at room temperature. Hitachi U1800 spectrophotometer (Hitachi, Tokyo, Japan) was used to read the absorbance at 415 nm. Rutin was used as the standard compound. Total flavonoid content was recorded as μg rutin equivalent (RE) g^− 1^ dw.

### Estimation of total antioxidant capacity (TAC)

The diphenyl-picrylhydrazyl (DPPH) radical degradation method [31] was used to estimate the antioxidant activity. 10 μl of leaf extract solution, 4 ml of distilled water and 1 ml of 250 μM DPPH solution were placed in a test tube. The tube was mixed thoroughly and allowed to stand for 30 min in the dark. Hitachi U1800 spectrophotometer (Hitachi, Tokyo, Japan) was used to read the absorbance at 517 nm. The antioxidant activity (ABTS^+^) was measured following the method of Sarker & Oba [31]. Separately, 7.4 mM ABTS^+^ solution and 2.6 mM potassium persulfate solution were prepared to make two different stock solutions. Two stock solutions were mixed in equal quantities to prepare the working solution and allowed to react for 12 h at room temperature in the dark. A 2850 μl of ABTS^+^ solution (1 ml ABTS^+^ solution mixed with 60 ml methanol) was allowed to react with 150 μl of leaf extract for 2 h in the dark. Hitachi U1800 spectrophotometer (Hitachi, Tokyo, Japan) was used to read the absorbance at 734 nm. The antioxidant activity was calculated as the percent of inhibition of DPPH and ABTS^+^ relative to the control using the following equation:

$$ \mathrm{Antioxidant}\ \mathrm{activity}\ \left(\%\right)=\left(\mathrm{Absorbance}\ \mathrm{of}\ \mathrm{blank}\hbox{-} \mathrm{Absorbance}\ \mathrm{of}\ \mathrm{sample}/\mathrm{Absorbance}\ \mathrm{blank}\right)\times 100 $$

Where, Absorbance blank is the absorbance of the control reaction [10 μl methanol for TAC (DPPH), 150 μl methanol for TAC (ABTS^+^) instead of leaf extract] and Absorbance sample is the absorbance of the test compound. Trolox was used as the reference standard, and the results were expressed as μg trolox equivalent g^− 1^ dw.

### Extraction of samples for HPLC and LC-MS analysis

1 g of leaves were homogenized with 10 ml of 80% methanol containing 1% acetic acid and filtered through a 0.45 μm filter using a MILLEX®-HV syringe filter (Millipore Corporation, Bedford, MA, USA) by the method of Khanam et al. [33]. The filtrate was centrifuged at 10,000 g for 15 min and used to analyze phenolic acids and flavonoids.

### HPLC analysis of phenolic acids and flavonoids

HPLC method described by Khanam et al. [33] was used to estimate the amounts of phenolic acids and flavonoids in amaranth leaves. LC-10Avp binary pumps, a degasser (DGU-14A) and a variable Shimadzu SPD-10Avp UV–vis detector were equipped to the HPLC system (Shimadzu SCL10Avp, Kyoto, Japan). Phenolic acids and flavonoids were separated through a CTO-10 AC (STR ODS-II, 150 × 4.6 mm I.D., Shinwa Chemical Industries, Ltd., Kyoto, Japan) column. The binary mobile phase consisted of 6% (*v*/v) acetic acid in water (solvent A) and acetonitrile (solvent B) was pumped at a flow rate of 1 ml/min for a total run time of 70 min. The system was run with a gradient program: 0–15% B for 45 min, 15–30% B for 15 min, 30–50% B for 5 min and 50–100% B for 5 min. The injection volume was 10 ml while the column temperature was maintained at 35 °C. For simultaneous monitoring of hydroxybenzoic acids, hydroxycinnamic acids and flavonoids, the detector was set at 254, 280 and 360 nm, respectively. The retention time and UV–vis spectra with those of standards were used to compare the identified compound. Mass spectrometry was also used to confirm the phenolic acids and flavonoids qualitatively. The sum of contents of HPLC quantified phenolic acids and flavonoids was denoted as the total phenolic index (TPI). Khanam et al. [33] method was used to obtain TPI. In each estimation, two samples were prepared for all analysis. The results were recorded as μgg^− 1^ fresh weight (FW).

The Mass spectrometry analyses were performed in negative ion mode using a JEOL AccuTOF (JMS-T100LP, JEOL Ltd., Tokyo, Japan) mass spectrometer. It was then fitted with an Agilent 1100 Series HPLC system and a UV–vis detector coupled on-line with an ElectroSpray Ionization (ESI) source. The column elute were recorded in the range of m/z 0–1000. Needle voltage was kept at − 2000 V. To obtain chromatograms with good resolution of adjacent peaks, a slight modification was made in the method reported by Khanam et al. [33] by optimizing the chromatographic conditions. LC-MS-ESI analysis was used to identify extract constituents.

### Statistical analysis

The results were described as the mean ± SD of three samples (*n* = 3) in each replication. Analysis of variance was performed by using Statistix 8 software. The mean separation was compared by the Duncan’s Multiple Range Test (DMRT) at 1% level of probability.

## Results

### Influence of nutritional compositions to drought stress

**Fig. 1:**
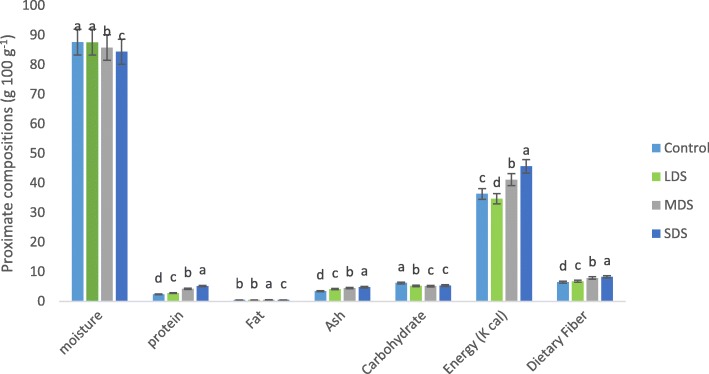
Changes of proximate compositions (g 100 g^− 1^) at four drought levels: Control (100% FC), LDS (90% FC), MDS (60% FC), and SDS (30% FC) in a selected *A. tricolor* genotype; (*n* = 3), letters mentioned in the bars are significantly varied by DMRT (*P* < 0.01)

**Fig. 2:**
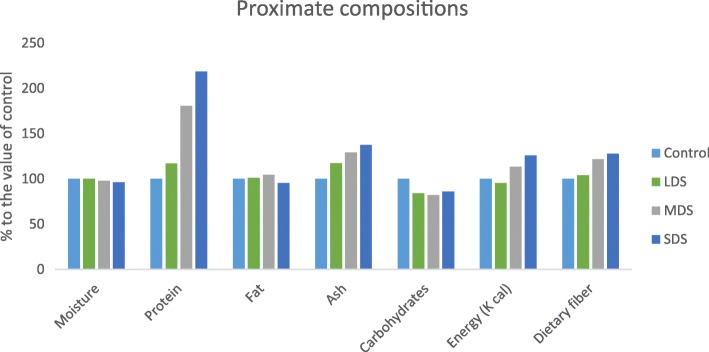
Effect of proximate composition (g 100 g^− 1^) (% to the value of control) at four drought levels: Control (100% FC), LDS (90% FC), MDS (60% FC), and SDS (30% FC) in a selected *A. tricolor* genotype

Effects of nutritional compositions under different drought stress of *A. tricolor* are presented in Fig. 1. Control and low drought stress (LDS) condition exhibited the highest moisture content, while the moisture content was gradually decreased from moderate drought stress (MDS) to severe drought stress (SDS). Moisture content was drastically reduced with the increase of drought stress in the order: (control > LDS > MDS > SDS). SDS condition had the highest protein, ash, and dietary fiber content, while the lowest protein, ash, and dietary fiber content were observed under the control condition. Protein, ash and dietary fiber content were remarkably increased with an increase in the severity of drought stress in the following order: control < LDS < MDS < SDS. In LDS, MDS and SDS, protein, ash and dietary fiber content were augmented by (17, 17 and 4%); (80, 29 and 21%) and (118, 38 and 28%); respectively over control condition (Fig. 2).

MDS condition had the highest fat content, and the lowest fat content was recorded at SDS condition, while the intermediate fat content was noticed under control and LDS conditions. Control condition had the highest carbohydrates content and it was gradually decreased in the order: control > LDS > MDS = SDS, which was statistically similar to MDS and SDS, respectively. Carbohydrate content was sharply declined with the severity of drought stress. The energy ranged from 34.67 to 45.61 g 100 g^− 1^ with the highest energy was recorded in SDS and the lowest in LDS condition.

### Drought stress effects on mineral content

**Fig. 3:**
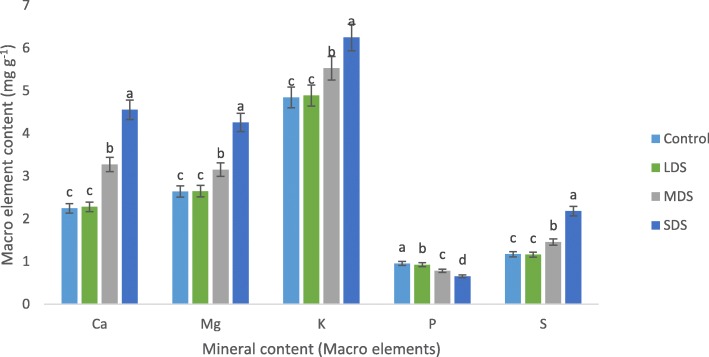
Response of mineral content (Macro elements mg g^− 1^) at four drought levels: Control (100% FC), LDS (90% FC), MDS (60% FC), and SDS (30% FC) in a selected *A. tricolor* genotype; (n = 3), letters mentioned in the bars are significantly varied by DMRT (*P* < 0.01)

**Fig. 4:**
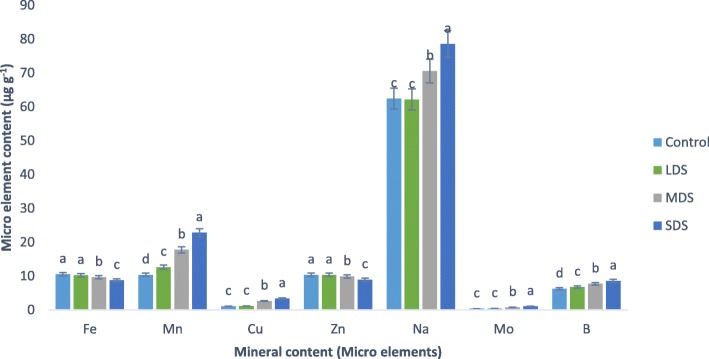
Impact of mineral content (Micro elements μg g^− 1^) at four drought levels: Control (100% FC), LDS (90% FC), MDS (60% FC), and SDS (30% FC) in a selected *A. tricolor* genotype; (*n* = 3), letters mentioned in the bars are significantly varied by DMRT (*P* < 0.01)

Results of minerals (macro and microelements) contents are presented in Figs. 3, 4. The mineral contents of *A. tricolor* were progressively influenced by drought stress. Ca, K, Na, Mg, S, Cu and Mo content were statistically similar under control and LDS conditions, whereas Ca, Mg, K, S, Cu, Na and Mo content were sharply and remarkably augmented with the severity of drought stress from MDS and SDS conditions showing the order: control = LDS < MDS < SDS.

**Fig. 5:**
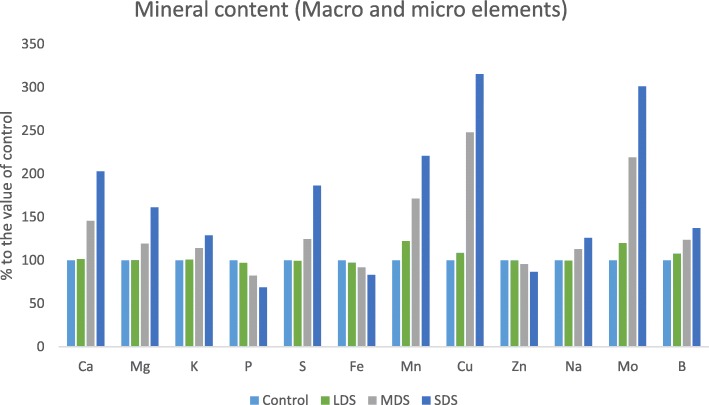
Assessment of mineral contents (Macro and microelements, mg g^− 1^ and μg g^− 1^, respectively) (% to the value of control) at four drought levels: Control (100% FC), LDS (90% FC), MDS (60% FC), and SDS (30% FC) in a selected *A. tricolor* genotype

In MDS and SDS, Ca, K, S, Mg, Na, Cu and Mo content were augmented by (46, 19, 14, 25, 148, 13 and 119%) and (103, 61, 29, 86, 215, 26 and 200%), respectively compared to control and LDS conditions (Fig. 5). B and Mn content were statistically increased with the increase of drought stress in the order: control <LDS < MDS < SDS. In LDS, MDS and SDS, the rate of increase of Mn and B were (22%, 8%), (71%, 23%) and (121%, 37%), respectively, over control condition (Fig. 5). Further, it was noted that, increasing drought stress lead to a significant decrease in P content in the following order: control > LDS > MDS > SDS. In LDS, MDS and SDS, reduction of P was 3%, 18% and 32%, respectively, over control condition (Fig. 5). Statistically, there were no significant differences in Fe and Zn content under control and LDS conditions, whereas Fe and Zn content were significantly and drastically declined with the severity of drought stress from MDS and SDS conditions showing the order: control = LDS > MDS > SDS. In MDS and SDS, Fe and Zn content were reduced by (8%, 5%) and (17%, 13%), respectively compared to control and LDS conditions (Fig. 5). The highest Ca, Mg, K, S, Mn, Cu, Na, Mo and B content were observed in SDS, while the lowest Ca, Mg, K, S, Mn, Cu, Na, Mo and B content were found in control or LDS condition. Conversely, the highest P, Fe and Zn content were recorded in control or LDS condition and the lowest P, Fe and Zn content were found in SDS.

### Drought stress effects on leaf pigments

**Fig. 6:**
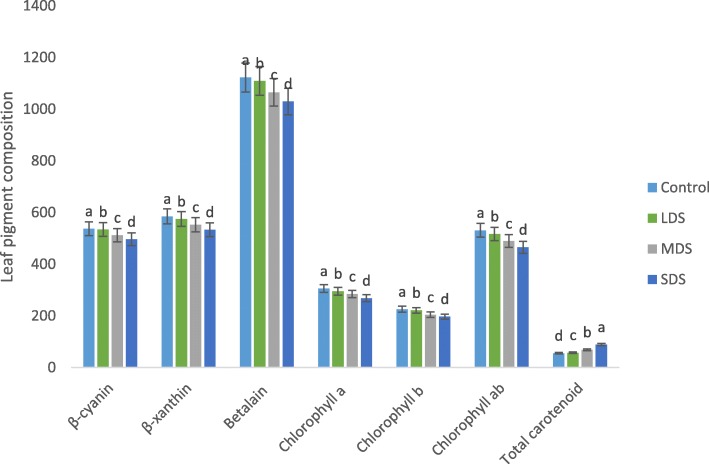
Influence of Leaf pigments at four drought levels: Control (100% FC), LDS (90% FC), MDS (60% FC), and SDS (30% FC) in a selected *A. tricolor* genotype; Betacyanin (ng g^− 1^ FW), Betaxanthin (ng g^− 1^ FW), Betalain (ng g^− 1^ FW), Chlorophyll a (μg g^− 1^ FW), Chlorophyll b (μg g^− 1^ FW), Chlorophyll ab (μg g^− 1^ FW), Total carotenoids (mg 100 g^− 1^ FW); (*n* = 3), letters mentioned in the bars are significantly varied by DMRT (*P* < 0.01)

**Fig. 7:**
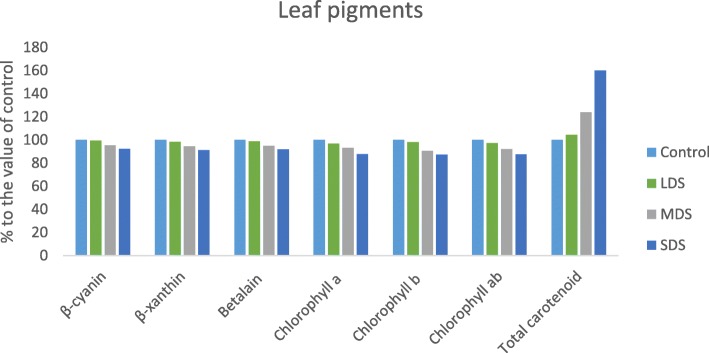
Comparison of leaf pigments (% to the value of control) at four drought levels: Control (100% FC), LDS (90% FC), MDS (60% FC), and SDS (30% FC) in a selected *A. tricolor* genotype; Betacyanin (ng g^− 1^ FW), Betaxanthin (ng g^− 1^ FW), Betalain (ng g^− 1^ FW), Chlorophyll a (μg g^− 1^ FW), Chlorophyll b (μg g^− 1^ FW), Chlorophyll ab (μg g^− 1^ FW),Total carotenoids (mg 100 g^− 1^ FW)

Leaf pigments of vegetable amaranth were significantly affected by drought stress (Fig. 6). Except total carotenoids, all the leaf pigments (Betacyanin, betaxanthin, betalain, chlorophyll *a*, chlorophyll *b* and chlorophyll *ab* content) were significantly and gradually reduced with the increase of the severity of drought stress (control > LDS > MDS > SDS). In LDS, MDS and SDS, betacyanin, betaxanthin, betalain, chlorophyll *a*, chlorophyll *b* and chlorophyll *ab* content were declined by (0.5, 2, 1, 3, 2 and 3%); (5, 5, 5, 7, 9 and 8%) and (8, 9, 9, 12, 12 and 12%); respectively, over control condition (Fig. 7). Betacyanin, betaxanthin, betalain, chlorophyll *a*, chlorophyll *b* and chlorophyll *ab* content were the highest in control condition, whereas betacyanin, betaxanthin, betalain, chlorophyll *a*, chlorophyll *b* and chlorophyll *ab* content were the lowest in SDS.

Total carotenoids were significantly and remarkably increased with the increasing the severity of drought stress (control < LDS < MDS < SDS). In LDS, MDS and SDS, total carotenoids were significantly and remarkably increased by 4, 24 and 60%, respectively over control condition (Fig. 7).

### Influence of betacarotene, vitamin C, TPC, TFC and TAC to drought stress

**Fig. 8:**
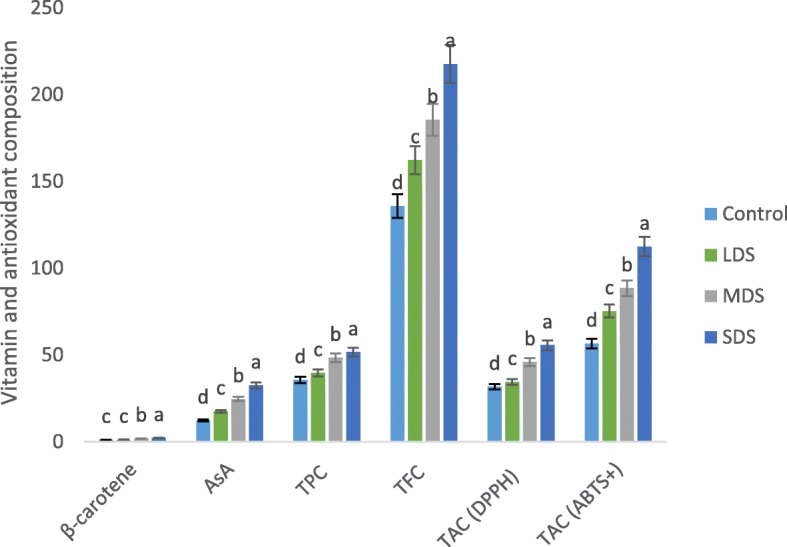
Response of Betacarotene, Vitamin C, TPC, TFC and TAC at four drought levels: Control (100% FC), LDS (90% FC), MDS (60% FC), and SDS (30% FC) in a selected *A. tricolor* genotype; AsA, Vitamin C (mg 100 g^− 1^); Betacarotene (mg g^− 1^), TFC, Total flavonoid content (RE μg g^− 1^ dw); TPC, Total polyphenol content (GAE μg g^− 1^ dw); TAC (ABTS^+^), Total antioxidant capacity (ABTS^+^) (TEAC μg g^− 1^ dw); TAC (DPPH), Total antioxidant capacity (DPPH) (TEAC μg g^− 1^ dw); (*n* = 3), letters mentioned in the bars are significantly varied by DMRT (*P* < 0.01)

**Fig. 9:**
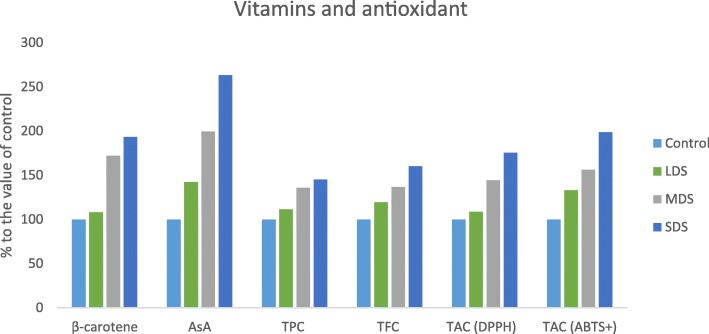
Response of Vitamins, TFC, TPC and TAC, (% to the value of control) at four drought levels: Control (100% FC), LDS (90% FC), MDS (60% FC), and SDS (30% FC) in a selected *A. tricolor* genotype; AsA, Vitamin C (mg 100 g^− 1^); Betacarotene (mg g^− 1^), TFC, Total flavonoid content (RE μg g^− 1^ dw); TPC, Total polyphenol content (GAE μg g^− 1^ dw); TAC (ABTS^+^), Total antioxidant capacity (ABTS^+^) (TEAC μg g^− 1^ dw) TAC (DPPH), Total antioxidant capacity (DPPH) (TEAC μg g^− 1^ dw)

Betacarotene, vitamin C content, TPC, TFC and TAC of *A. tricolor* were progressively influenced by drought stress Fig. 8. In this investigation, betacarotene, vitamin C content, total polyphenol content (TPC), total flavonoid content (TFC), total antioxidant capacity (TAC) (DPPH) and TAC (ABTS^+^) were significantly increased with the increasing of the severity of drought stress in the order: control < LDS < MDS < SDS. In LDS, MDS and SDS, betacarotene, vitamin C content, TPC, TFC, TAC (DPPH) and TAC (ABTS^+^) were augmented by (8, 42, 11, 19, 9 and 33%); (72, 100, 36, 37, 45 and 56%) and (93, 63, 45, 60, 75 and 99%); respectively, compared to control condition (Fig. 9). SDS condition had the highest betacarotene, vitamin C, TPC, TFC, TAC, (DPPH) and TAC (ABTS^+^), while the control condition exhibited the lowest betacarotene, vitamin C, TPC, TFC, TAC (DPPH) and TAC (ABTS^+^).

### Influence of drought stress on phenolics and flavonoids

Results of retention time, λmax, molecular ion, main fragment ions in MS^2^ and tentative compound identification for phenolic compounds are presented in Table 1. The values of phenolic acids and flavonoids components of *A. tricolor* genotype VA3 were separated though LC by comparing with masses of ion of standard flavonoids and phenolic acids and also by detecting the specific peaks of the corresponding components. A total of sixteen phenolic compounds were identified including six hydroxybenzoic acids, seven hydroxycinnamic acids and three flavonoids. *Trans*-cinnamic acid was newly identified phenolic acid in *A. tricolor*. However, an attempt was made for the first time to study the effect of drought stress in antioxidant enriched and high yield potential *A. tricolor* genotype VA3, in terms of sixteen phenolic acids and flavonoids. Within phenolic acids and flavonoids, hydroxybenzoic acids were identified as most abundant compounds in this genotype. Among hydroxybenzoic acids, salicylic acid was identified as one of the main phenolic acids followed by vanilic acid and gallic acid and *p*-hydroxybenzoic acid. Considering hydroxycinnamic acids, chlorogenic acid and *Trans*-cinnamic acid were the most abundant compound followed by *m*-coumaric acid. A good amount of caffeic acid, *p*-coumaric acid and ferulic acid were also identified in this genotype. In this investigation, the flavonoids, rutin (quercetin-3-rutinoside) and isoquercetin (quercetin-3-glucoside) were the most abundant in this genotype.

**Table 1:** Retention time (Rt), wavelengths of maximum absorption in the visible region (λ_max_), mass spectral data, tentative identification of phenolic compounds and quantification (μgg^−1^ FW) in *Amaranthus tricolor* leaves

Phenolic compound	Rt (min)	λ_max_ (nm)	Molecular ion [M - H]^−^ (m/z)	Identity	MS^2^ (m/z)	Control (100% FC)	LDS (90% FC)	MDS (60% FC)	SDS (30% FC)
Hydroxybenzoicacid									
Gallic acid	9.1	254	169	3,4-5Trihydroxybenzoicacid	169.2	7.23 ± 0.03d	8.64 ± 0.04c	10.25 ± 0.05b	12.25 ± 0.06a
Vanilic acid	30.6	254	167	4-hydroxy-3-methoxybenzoicacid	167.2	9.75 ± 0.07d	10.12 ± 0.05c	12.83 ± 0.04b	15.48 ± 0.08a
Syringic acid	34.8	254	197	4-Hydroxy-3,5-dimethoxybenzoicacid	197.1	1.17 ± 0.02c	1.22 ± 0.01c	1.65 ± 0.02b	1.83 ± 0.01a
*p*-hydroxybenzoic acid	31.5	254	137	4-hydroxybenzoic acid	137.2	2.64 ± 0.03d	2.84 ± 0.02c	3.26 ± 0.02b	4.07 ± 0.03a
Salicylic acid	48.2	254	137	2-Hydroxybenzoic acid	137.2	17.45 ± 0.21d	18.96 ± 0.12c	25.68 ± 0.14b	28.96 ± 0.16a
Ellagic acid	52.5	254	301	(2,3,7,8-tetrahydroxy-chromeno [5,4,3-cde]chromene-5,10-dione	301.1	0.98 ± 0.01d	1.04 ± 0.02c	2.15 ± 0.02b	2.08 ± 0.03a
Total benzoic acids						39.22	42.81	55.81	64.66
Hydroxycinnamic acid									
Caffeic acid	32.0	280	179	3,4-Dihydroxy-trans-cinnamate	179.1	1.56 ± 0.02d	1.68 ± 0.01c	1.96 ± 0.02b	2.68 ± 0.03a
Chlorogenic acid	31.1	280	353	3-(3,4-Dihydroxycinnamoyl) quinic acid	353.2	9.86 ± 0.18d	10.26 ± 0.24c	12.54 ± 0.26b	13.86 ± 0.20a
*p*-coumaric acid	42.0	280	163	4-hydroxycinnamicacid	163.1	1.04 ± 0.02d	1.12 ± 0.01c	2.14 ± 0.02b	2.24 ± 0.02a
Ferulic acid	47.9	280	193	4-hydroxy-3-methoxycinnamicacid	193.2	1.02 ± 0.01c	1.08 ± 0.02c	1.55 ± 0.01b	2.15 ± 0.03a
*m*-coumaric acid	49.6	280	163	3-hydroxycinnamicacid	163.3	3.13 ± 0.03d	3.54 ± 0.02c	6.55 ± 0.04b	7.96 ± 0.05a
Sinapic acid	49.0	280	223	4-Hydroxy-3,5-dimethoxycinnamicacid	223.2	0.26 ± 0.01d	0.34 ± 0.01c	0.38 ± 0.01b	0.42 ± 0.01a
*Trans*-cinnamic acid	67.3	280	147	3-Phenylacrylic acid	147.1	5.03 ± 0.02d	5.26 ± 0.01c	5.54 ± 0.01b	5.65 ± 0.02a
Total cinnamic acids						21.89	23.27	31.66	34.96
Flavonoids									
Iso-quercetin	54.3	360	463	Quercetin-3-glucoside	463.3	3.55 ± 0.02c	3.58 ± 0.03c	6.46 ± 0.02b	7.82 ± 0.04a
Hyperoside	53.3	360	463	Quercetin-3-galactoside	463.5	1.18 ± 0.01c	1.22 ± 0.02c	1.58 ± 0.01b	2.05 ± 0.02a
Rutin	53.0	360	609	Quercetin-3-rutinoside	609.4	7.89 ± 0.06c	7.96 ± 0.05c	9.58 ± 0.06b	11.24 ± 0.04a
Total flavonoids						57.11	62.08	82.47	95.62
Total phenolic acids						16.59	18.76	21.62	25.12
Total phenolic index						73.70	78.84	104.09	120.74

Different letters in a row are differed significantly by Duncan Multiple Range Test (*P* < 0.01); (*n* = 3)

**Fig. 10:**
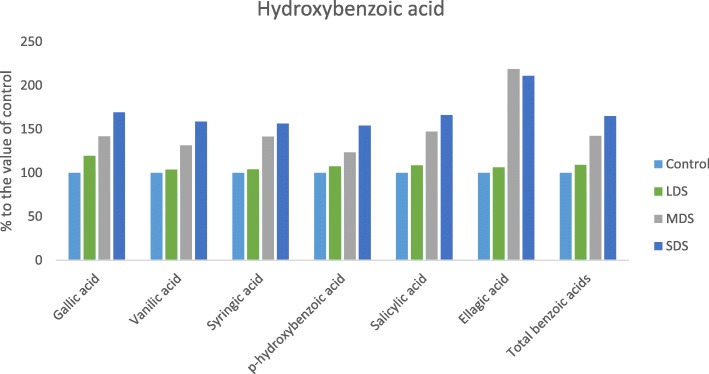
Changes of hydroxybenzoic acid compositions (μg g^− 1^FW) (% to the value of control) at four drought levels: Control (100% FC), LDS (90% FC), MDS (60% FC), and SDS (30% FC) in a selected *A. tricolor* genotype

**Fig. 11:**
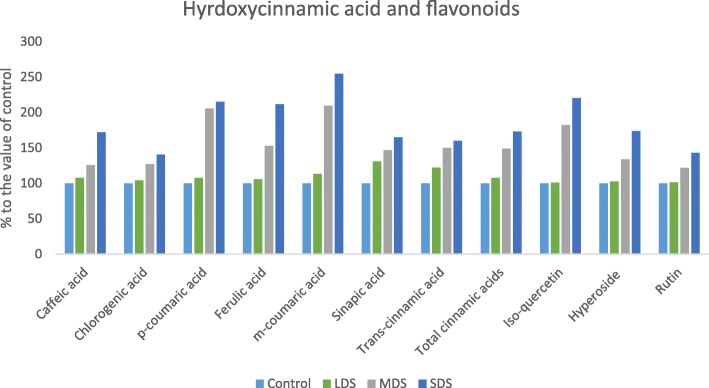
Changes of hydroxycinnamic acid and flavonoid compositions (μg g^− 1^FW) (% to the value of control) at four drought levels: Control (100% FC), LDS (90% FC), MDS (60% FC), and SDS (30% FC) in a selected *A. tricolor* genotype

The hydroxybenzoic acid (Syringic acid and); the hydroxycinnamic acid (Ferulic acid) and three flavonoids, iso-quercetin, hyperoside and rutin had no significant differences in their compositions under control and LDS conditions, nevertheless, the composition of these acids were significantly increased from MDS to SDS. In MDS and SDS, these phenolic acids and flavonoids compositions were increased by (41, 53, 82 34 and 22%) and (56, 111, 121 74 and 43%); respectively compared to control or LDS condition (Figs. 10, 11). Five hydroxybenzoic acids (Gallic acid, vanilic acid, *p*-hydroxybenzoic acid, salicylic acid and ellagic acid) and six hydroxycinnamic acid (Caffeic acid, chlorogenic acid, *trans*-cinnamic acid, *p-*coumaric acid, *m-*coumaric acid and sinapic acid) were remarkably increased with the increment of the severity of drought stress in the order: Control< LDS < MDS < SDS. In LDS, MDS and SDS, these phenolic acids and flavonoids concentrations were increased by (19, 4, 7, 9 6, 8, 4, 8, 13, 31 and 22%); (42, 32, 23, 19, 42, 26, 27, 105, 109, 47 and 50%) and (69, 59, 54, 66,111, 65, 72, 41, 115, 45, 154, 65 and 60%); respectively (Figs.10, 11).

## Discussion

As leafy vegetables, *A. tricolor* leaves exhibited high moisture content. Nevertheless, it demonstrated a noble source of protein, dietary fiber, carbohydrates and ash. Moisture content was significantly reduced with the increment of drought stress in the following order: (control > LDS > MDS > SDS). As lower moisture contents of leaves ensured higher dry matter, the drought-stressed plant could be a promising source of dry matter compared to control condition. In LDS, MDS and SDS, protein, ash and dietary fiber content were augmented by (17, 17 and 4%); (80, 29 and 21%) and (118, 38 and 28%); respectively over control condition. However, Siracusa et al. [17] observed a decrease in protein content at drought stress to fully irrigated in buckwheat. The genotypic variances between two crops might be contributed for the different results. *A. tricolor* is the sources of protein for vegetarian and poor people of the third world countries. Dietary fiber has a significant role in palatability, digestibility and remedy of constipation [22]. MDS condition had the highest fat content, and the lowest fat content was observed under SDS condition. Fats are sources of omega-3 and omega-6 fatty acids. It helps in the digestion, absorption, and transport of fat-soluble vitamins A, D, E, and K. Sun et al. [34] observed similar results in sweet potato leaves where they mentioned that fat involved in the insulation of body organs and in the maintenance of body temperature and cell function. Control had the highest carbohydrates content and it was gradually decreased in the order: control > LDS > MDS = SDS, which was statistically similar to MDS and SDS, respectively. Carbohydrate content sharply declined with the severity of drought stress. As a leafy vegetable, the low carbohydrate content of amaranth leaves has no a substantial effect on the daily diet of human body. As regards energy balance, SDS exhibited remarkably higher calories compared to MDS, LDS and control conditions, while these variations have no remarkable impact on the daily diet of the human body, since very little amounts were consumed in the daily diet. Drought stress increased the protein, ash, energy, fat and dietary fiber content and reduced carbohydrate and moisture content of *Amaranthus* leaves. For this, *Amaranthus* leafy vegetable might be produced in a semi-arid and dry area in the world could be contributed as a noble source of dietary fiber and vegetarian protein in human diet.

Amaranth leaves are noble sources of minerals (macro and microelements). The mineral content of amaranth leaves was remarkably influenced by drought stress. Zinc and Fe content of *A. tricolor* are greater than that of the cassava leaves [35] and beach pea [36]. Similarly, Jimenez-Aguiar & Grusak [37] reported high Fe, Mn, Cu and Zn (fresh weight basis) in different *A. spp.* including *A. tricolor*. They also reported that Amaranths had higher Zn content than black nightshade, spinach and kale; more Fe and Cu content than kale. Ca, Mg, K, S, Cu, Na and Mo content were sharply and remarkably augmented with the severity of drought stress from MDS and SDS conditions showing the order: control = LDS < MDS < SDS. On the other hand, Hanson et al. [14] reported a decline in Ca content both in Choysum and Kailaan varieties from dry season to wet season trial. SDS exhibited the highest Ca, K, S, Mg, Mn, Na, Cu, Mo and B content, while control or LDS condition had the lowest Ca, S, K, Mg, Cu, Mn, Mo, Na and B content. In contrast, control or LDS condition had the highest Zn, P, and Fe content and SDS exerted the lowest P, Zn and Fe content. Likewise, Hanson et al. [14] recorded a decline in Fe content of Choysum variety, whereas they reported a sharp increment in Kailaan variety from dry season to wet season trial. Moreover, Hanson et al. [14] recorded a remarkable increment in Zn content both in Kailaan and Choysum variety from dry season to wet season trial. Except P, Fe and Zn, all the mineral content were progressively raised with the increment of drought stress, whereas, Zn, Fe and P were sharply declined with the increment of drought stress. Therefore, *A. tricolor* cultivated in a drought-stressed area specifically in semi-arid and drought-prone area could be contributed as a noble source of minerals in the daily diet of human body related to usual farming practices.

Leaf pigments of *A. tricolor* were statistically influenced by drought stress. Except total carotenoids, all the leaf pigments (Betacyanin, betaxanthin, betalain, chlorophyll *a*, chlorophyll *b* and chlorophyll *ab* content) were significantly and gradually reduced with the increasing the severity of drought stress (control > LDS > MDS > SDS). Likewise, Hsu and Kao [38] reported a decline in chlorophyll content with the increment of drought severity. They also stated that drought stress influenced growth and development of plant through osmotic stress, declining the water potential, reducing stomatal conductivity which limits CO_2_ influx to leaves and unfavorable CO_2_/O_2_ ratio in chloroplasts, decreasing photosynthesis.

Betacarotene, vitamin C content, TPC, TFC and TAC of *A. tricolor* were progressively influenced by drought stress. In this investigation, betacarotene, vitamin C content, total polyphenol content (TPC), total flavonoid content (TFC), total antioxidant capacity (TAC) (DPPH) and TAC (ABTS^+^) were significantly increased with the increase of the severity of drought stress in the order: control < LDS < MDS < SDS. SDS condition had the highest betacarotene, vitamin C, TPC, TFC, TAC, (DPPH) and TAC (ABTS^+^), while the control condition exhibited lowest betacarotene, vitamin C, TPC, TFC, TAC (DPPH) and TAC (ABTS^+^). Hanson et al. [14] reported an increase in betacarotene content of Choysum variety. In contrast, they reported a declining trend in betacarotene content of Kailaan variety and reduction in vitamin C content in both varieties from dry to wet season trial. The reason for reduction might be due to the genotypic variations in two different crops. Likewise, Gharibi et al. [18] in *Achillea* species and Siracusa et al. [17] in buckwheat, reported increment in antioxidant activity, polyphenol and flavonoid content with the severity of drought stress. The ameliorate response of betacarotene content with the severity of drought stress was also reported in Choysum varieties in dry season trial [14], and in perennial herbaceous [15]. Sarker and Oba [39] observed increase in betacarotene content with the severity of salt stress. Siracusa et al. [17] reported an increment of TPC, TFC in buckwheat with increasing the drought stress. Garibi et al. [18] also reported the enhancing response of TPC, TFC and antioxidant activity in *Achillea* species with the increment of drought stress.

A total of sixteen phenolic compounds were identified including six hydroxybenzoic acids, seven hydroxycinnamic acids and three flavonoids. *Trans*-cinnamic acid was newly identified phenolic acid in *A. tricolor*. Khanam & Oba [40] in red and green amaranths and Khanam et al. [33] in eight different leafy vegetables including amaranths described rest fifteen phenolic acids and flavonoids with normal cultivation practices. However, an attempt was made for the first time to study the effect of drought stress in high yielding and high antioxidant containing *A. tricolor* cultivar VA3, in terms of sixteen phenolic acids and flavonoids. Gallic acid, vanilic acid and *p*-hydroxybenzoic acid content of the genotype VA3 under control condition were higher than *A. tricolor* genotypes that reported by Khanam et al. [33]. Considering hydroxycinnamic acids, chlorogenic acid and *trans*-cinnamic acid were the most abundant compound followed by *m*-coumaric acid. A good amount of caffeic acid, *p*-coumaric acid and ferulic acid were also identified in this genotype. Under control condition, chlorogenic acid, caffeic acid and *m*-coumaric acid of this genotype was higher than *A. tricolor* genotypes reported by Khanam et al. [33]. In plant tissues, phenylalanine produced widely dispersed phenolic acids i. e., hydroxycinnamic acids [41]. The most common forms of flavonoids are glycoside derivatives even though these compounds occasionally occur as a glycone in plants. Flavonoids represent 60% of total dietary phenolic compounds [42]. In plants, most predominant flavonoids are flavonols and the most prevalent naturally occurring flavonols are glycosides of quercetin [42]. We observed that isoquercetin (quercetin-3-glucoside) and rutin (quercetin-3-rutinoside) were the most abundant favonoids in VA3. Our study demonstrated that VA3 revealed higher rutin (quercetin-3-rutinoside) content at normal cultural practices compared to *A. tricolor* genotypes reported by Khanam et al. [33]. All the phenolic acids and flavonoids had the lowest concentrations under control condition, whereas these acids exhibited the highest concentrations under SDS condition. Hence, *A. tricolor* cultivated in a drought-stressed area specifically in the semi-arid and drought-prone area could be contributed as a noble source of minerals and bioactive compounds, phenolics and flavonoid content and antioxidant activity in the daily diet of human body related to usual farming practices.

## Conclusions

In this study, antioxidant enriched and high yield potential *A. tricolor* genotype VA3 was selected from our germplasm collection and evaluated for nutritional and bioactive compounds, phenolic acids, flavonoids and antioxidant capacity under 4 irrigation regimes. *Trans*-cinnamic acid was newly identified phenolic acid in *A. tricolor*. Drought stress resulted in significant increment in protein, ash, energy, dietary fiber, K, S, Ca, Mn, Mg, Na, Cu, Mo and B content, total carotenoids, betacarotene, vitamin C, TAC (DPPH), TFC, TPC and TAC (ABTS^+^), sixteen phenolic acids and flavonoids. All the nutritional and bioactive compounds, phenolics, flavonoids and antioxidant capacity of *A. tricolor* leaves was very high under MDS and SDS condition, in comparison to control condition, that could be contributed as valuable food sources for human diets and health benefit. Nutritional and bioactive compounds, phenolics, flavonoids might be played a vital role in scavenging ROS and would be beneficial for human nutrition by serving as good antioxidants and antiaging sources in human health benefit. Moreover, *A. tricolor* cultivated under drought stress could be contributed as a quality product of nutritional and bioactive compounds, phenolics, flavonoids and antioxidants. Based on the results reported farmers of semi-arid and dry areas of the world could be able to grow amaranth as an alternative crop.

## Abbreviations

AAS: Atomic absorption spectrophotometry
ABTS^+^: 2,2′-azino-bis-(3-ethylbenzothiazoline-6-sulphonic acid)
AEZ: Agro-ecological zone
AOAC: Association of analytical communities
AsA: vitamin C
ASAE: American society of association executives
B: Boron
Ca: Calcium
Cu: Copper
DHA: Dehydroascorbate
DPPH: 2,2-diphenyl-1-picrylhydrazyl
DTT: Dithiothreitol
dw: Dry weight
FC: Field capacity
Fe: Iron
FW: Fresh weight
H_2_SO_4_: Sulphuric acid
HClO_4_: Perchloric acid
HNO_3_: Nitric acid
HPLC-UV: High performance liquid chromatography-ultra violet ISO International organization for standardization
K: Potassium
LC-MS-ESI: Liquid chromatography-mass spectroscopy-electrospray ionization
LDS: Low drought stress
MDS: Moderate drought stress
Mg: Magnesium
Mn: Manganese
Mo: Molybdenum
Na: Sodium
NaOH: Sodium hydroxide
P: Phosphorus
ROS: Reactive oxygen species
S: Sulphur
SDS: Severe drought stress
TAC: Total antioxidant capacity
TFC: total flavonoid content
TPC: Total polyphenol content
Zn: Zinc
